# Impact of Chronic Kidney Disease on Contrast-Induced Nephropathy, Bleeding, and Clinical Outcomes After Rotational Atherectomy: A Multicenter Retrospective Study

**DOI:** 10.3390/medicina62030597

**Published:** 2026-03-21

**Authors:** Jaeyun Lee, Jin Jung, Sang-Suk Choi, Sung-Ho Her, Kyusup Lee, Ki-Dong Yoo, Keon-Woong Moon, Donggyu Moon, Su-Nam Lee, Won-Young Jang, Ik-Jun Choi, Jae-Hwan Lee, Jang-Hoon Lee, Sang-Rok Lee, Seung-Whan Lee, Kyeong-Ho Yun, Hyun-Jong Lee

**Affiliations:** 1 Department of Nephrology, St. Vincent’s Hospital, College of Medicine, The Catholic University of Korea, Seoul 16247, Republic of Korea; jaeyun0520@gmail.com; 2 Department of Cardiology, St. Vincent’s Hospital, College of Medicine, The Catholic University of Korea, Seoul 16247, Republic of Korea; colaking@naver.com (J.J.); yookd@catholic.ac.kr (K.-D.Y.); cardiomoon@gmail.com (K.-W.M.); babaheesu@gmail.com (D.M.); yellow-night@hanmail.net (S.-N.L.); raph83@naver.com (W.-Y.J.); 3 Catholic Research Institute for Intractable Cardiovascular Disease, College of Medicine, The Catholic University of Korea, Seoul 06591, Republic of Korea; ajobi7121@gmail.com (K.L.); mrfasthand@catholic.ac.kr (I.-J.C.); 4 Department of Cardiology, Daejeon St. Mary’s Hospital, College of Medicine, The Catholic University of Korea, Seoul 34943, Republic of Korea; 5 Department of Cardiology, Incheon St. Mary’s Hospital, College of Medicine, The Catholic University of Korea, Incheon 21431, Republic of Korea; 6 Department of Cardiology in Internal Medicine, Chungnam National University School of Medicine, Chungnam National University Sejong Hospital, Sejong 30099, Republic of Korea; myheart@cnuh.co.kr; 7 Department of Internal Medicine, Kyungpook National University Hospital, Daegu 41944, Republic of Korea; ljhmh75@knu.ac.kr; 8 Department of Cardiology, Jeonbuk National University Hospital, Jeonju 54907, Republic of Korea; medorche@naver.com; 9 Department of Cardiology, Asan Medical Center, University of Ulsan College of Medicine, Seoul 05505, Republic of Korea; seungwlee@amc.seoul.kr; 10 Department of Cardiovascular Medicine, Regional Cardiocerebrovascular Center, Wonkwang University Hospital, Iksan 54538, Republic of Korea; ards7210@wonkwang.ac.kr; 11 Department of Internal Medicine, Sejong General Hospital, Bucheon 14754, Republic of Korea; untouchables00@hanmail.net

**Keywords:** rotational atherectomy (RA), chronic kidney disease (CKD), contrast-induced nephropathy (CIN)

## Abstract

*Background and Objectives*: Chronic kidney disease (CKD) is associated with severe coronary calcification and increased procedural risks. We aimed to evaluate the impact of CKD on contrast-induced nephropathy (CIN), bleeding, and clinical outcomes in patients undergoing rotational atherectomy (RA). *Materials and Methods*: This study retrospectively analyzed 652 patients who underwent RA for calcified coronary lesions from the multicenter ROCK registry and a single-center extension between 2010 and 2025. Patients were classified into CKD (eGFR < 60 mL/min/1.73 m^2^, *n* = 66) and non-CKD (*n* = 586) groups, excluding those on dialysis. The primary endpoint was a composite of CIN and in-hospital bleeding. Secondary endpoints included 3-year target vessel failure (TVF), myocardial infarction (MI), and total bleeding. *Results*: The primary composite outcome occurred more frequently in the CKD group (16.7% vs. 5.1%, *p* = 0.001). Specifically, CIN was significantly higher in CKD patients (15.2% vs. 1.7%, *p* < 0.001), while in-hospital bleeding did not differ significantly. In multivariate analysis, CKD was an independent predictor of the primary outcome (adjusted OR 3.02; 95% CI 1.36–6.69; *p* = 0.006). At 3-year follow-up, total bleeding (10.6% vs. 3.9%, *p* = 0.008) and MI (6.1% vs. 2.1%, *p* = 0.024) were higher in the CKD group, whereas TVF and cardiac death showed no significant difference. *Conclusions*: CKD is a robust independent risk factor for CIN and long-term bleeding in patients undergoing RA. However, comparable clinical efficacy outcomes suggest that RA remains a feasible strategy in CKD patients when early complications are carefully managed with contrast-minimizing strategies.

## 1. Introduction

Chronic kidney disease (CKD) is associated not only with a higher burden of coronary artery disease but also with a disproportionately high prevalence of severe coronary artery calcification; a meta-analysis reported coronary artery calcification in approximately 60% of CKD patients, and observational data suggest that calcification can be present in up to 90% of patients with a more advanced CKD group [1,2,3].

Disturbances in mineral metabolism, particularly hyperphosphatemia and calcium–phosphate imbalance, induce the phenotypic transformation of vascular smooth muscle cells into osteogenic-like cells, leading to progressive vascular calcification [4,5]. In addition, chronic inflammation, oxidative stress, and the accumulation of uremic toxins further contribute to the progression of vascular calcification in patients with CKD [6,7].

Severe coronary calcification is a well-studied risk factor for procedural complexity and is associated with suboptimal percutaneous coronary intervention (PCI) performance and worse clinical outcomes, including higher rates of repeat revascularization and adverse events after intervention [8,9].

In patients with CKD, contrast using invasive procedures such as coronary angiography and PCI carry a heightened risk of contrast-induced nephropathy (CIN) and bleeding complications compared with patients with preserved renal function, reflecting both reduced renal reserve and a CKD-related bleeding diathesis [10]. Indeed, in the recent European Society of Cardiology (ESC) guidelines for acute and chronic coronary syndromes, CKD is classified as a condition associated with increased ischemic and bleeding risk, requiring careful risk stratification and individualized treatment strategies [11,12]. In addition, recent ESC/EAPCI-endorsed evidence highlights that heavily calcified coronary lesions represent a major determinant of procedural complexity and adverse outcomes in PCI, often requiring advanced lesion preparation strategies [13].

Rotational atherectomy (RA) is an established strategy for severely calcified, high-complexity coronary lesions; however, it is used in a relatively small proportion of PCI procedures worldwide, typically on the order of 1–2% of all PCI cases in national datasets [14,15]. Patients requiring advanced plaque modification such as RA are exposed to unique procedural factors beyond simple contrast exposure. RA induces mechanical hemolysis, micro-embolization of debris, and a significant systemic inflammatory response characterized by intense platelet activation [16,17]. These RA-specific factors may impair renal function, yet how these risks translate into clinical CIN and bleeding outcomes specifically in the CKD population remains poorly defined.

Despite the growing body of RA literature, data specifically evaluating CIN and bleeding, along with long-term clinical outcomes in RA-treated patients, particularly stratified by CKD status, remain limited. Therefore, we conducted a retrospective analysis based on an RA registry to compare CIN, bleeding, and clinical outcomes between CKD and non-CKD patients undergoing RA.

## 2. Materials and Methods

### 2.1. Study Design and Population

**Figure 1:**
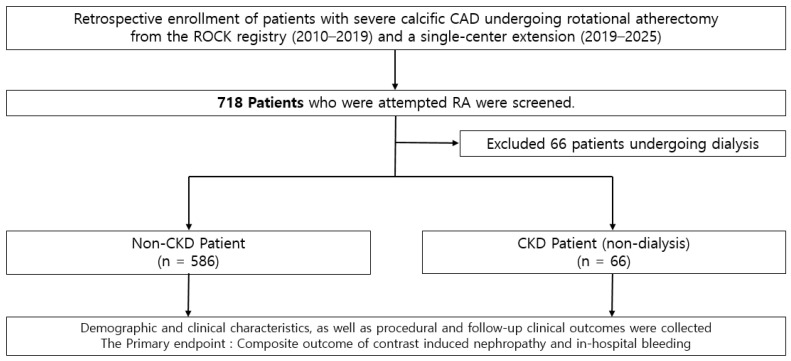
Study population and flow chart.

The clinical data of 540 patients with severe calcific coronary artery disease who underwent PCI using RA between January 2010 and October 2019 at nine tertiary centers from the ‘ROtational atherectomy in Calcified lesions in Korea (ROCK)’ registry and an additional 178 patients from Saint Vincent hospital from November 2019 to August 2025 were retrospectively analyzed. Among them, 66 patients under dialysis were excluded since our main outcome, CIN, was not able to be evaluated in dialysis patients. Clinical data from a total of 652 patients were collected at each participating center using a standardized case report form that included procedural information and follow-up outcomes. Follow-up data were retrospectively obtained through a review of medical records or, when necessary, through direct contact with patients or treating physicians. The study protocol was approved by the institutional review board of each participating center, and the requirement for informed consent was waived due to the retrospective nature of the study. All procedures were conducted in accordance with applicable ethical standards and relevant guidelines. From 2010 to 2019, consecutive patients with severe coronary calcification and significant luminal narrowing (≥70% diameter stenosis) who underwent PCI with rotational atherectomy were identified from institutional databases and included in the analysis. Chronic kidney disease was defined as an estimated glomerular filtration rate of <60 mL/min/1.73 m^2^, calculated using the Modification of Diet in Renal Disease equation based on baseline serum creatinine [18]. The study population and flow chart are described in Figure 1.

### 2.2. RA Procedure

Following the protocol of the ROCK registry, procedural strategies—including the indication and timing of rotational atherectomy (RA), burr size selection, and vascular access—were determined at the discretion of the treating interventional cardiologist, taking into account clinical risk profiles, lesion characteristics, and overall patient condition. All procedures were performed using standard interventional techniques. RA was conducted with the Rotablator™ system (Boston Scientific, Marlborough, MA, USA). During atherectomy, short and intermittent ablation runs were employed, and intracoronary vasodilators such as nitroglycerin and/or verapamil were administered to minimize the risk of coronary spasm and slow-flow phenomena. Antiplatelet therapy and peri-procedural anticoagulation were managed in accordance with current guideline recommendations [19,20]. Also, a direct RA strategy was defined as an early utilization of RA, including cases in which RA was performed (1) without prior balloon dilation, (2) after minimal pre-dilation using a balloon < 2.0 mm, (3) based on operator judgment indicating resistance to balloon passage, or (4) in the presence of severe calcification confirmed by imaging. In contrast, an indirect RA approach referred to RA performed following balloon dilation with a balloon size ≥ 2.0 mm.

For the prevention of contrast-induced nephropathy (CIN), a standardized institutional hydration protocol was applied. Routine intravenous hydration with normal saline at a rate of 1 mL/kg/h was administered before the procedure and maintained for 12 h post-procedure. In patients with a left ventricular ejection fraction (LVEF) ≤ 40% or a high risk of volume overload (e.g., history of heart failure), the hydration regimen was modified to 0.45% saline, or the infusion rate was individualized to avoid pulmonary edema. In addition, prophylactic oral N-acetylcysteine (600 mg twice daily) was routinely administered. Given the multicenter nature of the registry, there were no specific restrictions regarding the osmolarity of the contrast media used (e.g., low-osmolar or iso-osmolar). The selection of the contrast agent varied across participating institutions and was left to the operator’s discretion, based on the specific procedure and the patient’s clinical condition.

### 2.3. Study Outcomes

The primary endpoint of the study was a composite of CIN and in-hospital bleeding. And the individual outcome was also evaluated. CIN was defined as a deterioration in renal function, indicated by either a ≥25% increase in serum creatinine from baseline or an absolute increase of ≥0.5 mg/dL within 48–72 h after the procedure. Secondary endpoints included 3-year clinical outcomes, such as target vessel failure (TVF), defined as a composite of cardiac death, target vessel myocardial infarction (TVMI), or target vessel revascularization (TVR), as well as all-cause mortality, cardiac death, any myocardial infarction, TVMI, any repeat revascularization (RR), TVR, target lesion revascularization (TLR), stent thrombosis (ST), cerebrovascular accident (CVA), and bleeding events. Bleeding outcomes observed in this study were defined according to the Thrombolysis in Myocardial Infarction (TIMI) scale [21,22]. We have used the TIMI major and minor scale, and total bleeding was a composite of major and minor bleeding events. Technical success was defined as achieving residual stenosis <30% with a final TIMI grade III flow. Procedural success was defined as technical success without in-hospital major adverse cardiac and cerebrovascular events (MACCEs), including death, stroke, urgent revascularization (PCI or CABG), peri-procedural myocardial infarction, or stent thrombosis during hospitalization. Procedure-related complications included cardiac tamponade, coronary perforation, and severe coronary dissection (National Heart, Lung, and Blood Institute classification types D–F), as well as temporary pacemaker insertion, CIN, and in-hospital bleeding. Procedural characteristics such as total procedure time, radiation exposure, and contrast volume were assessed to evaluate procedural efficiency and safety. All-cause death was defined as death from any cause. TVMI referred to spontaneous myocardial infarction attributable to the treated vessel. Spontaneous myocardial infarction was defined as an elevation of cardiac biomarkers above the upper reference limit accompanied by ischemic symptoms or signs during follow-up after discharge. Peri-procedural myocardial infarction was defined as a peak creatine kinase–myocardial band level exceeding 10 times the upper reference limit within 48 h after the procedure [23]. Repeat revascularization was defined as any percutaneous or surgical revascularization in any vessel, while TVR and TLR were defined as revascularization of the target vessel and target lesion, respectively. Cerebrovascular accident was defined as a neurologically confirmed focal deficit of central origin lasting more than 24 h and supported by neurologist and imaging findings. All clinical events were verified using source documentation at each participating center and adjudicated by an independent clinical events committee blinded to the type of revascularization.

### 2.4. Statistical Analyses

Continuous variables are reported as mean ± standard deviation (SD) and were compared using either Student’s *t*-test or the Mann–Whitney U test, as appropriate. Categorical variables are expressed as frequencies with percentages and were analyzed using the chi-square test or Fisher’s exact test depending on the data distribution. The primary outcomes were analyzed using multivariable logistic regression models. Odds ratios with 95% confidence intervals were calculated to estimate the independent association between CKD status and the occurrence of each outcome after adjustment for predefined covariates. Event rates were evaluated using Kaplan–Meier survival analyses for time-to-first-events and compared between groups with the log-rank test. Univariate Cox proportional hazards models were initially applied to estimate hazard ratios (HRs) for clinical outcomes. Variables identified as clinically relevant or showing statistical significance (*p* < 0.05) in univariate analysis were subsequently included in multivariable Cox regression models to determine independent predictors of outcomes. For the secondary outcomes, to address potential model instability and overfitting arising from the limited number of clinical events, we employed Firth’s penalized likelihood Cox regression for multivariable analyses. This method was specifically used to provide more reliable hazard ratio estimates by reducing small-sample bias, ensuring the robustness of our findings in the presence of rare outcomes such as myocardial infarction. Subgroup analyses were conducted using Cox regression models, and the results were presented as forest plots. All statistical analyses were performed using SAS version 9.4 (SAS Institute Inc., Cary, NC, USA), with statistical significance defined as a two-sided *p*-value < 0.05.

## 3. Results

### 3.1. Baseline Characteristics

Baseline characteristics are summarized in Table 1, Table 2 and Table 3. A total of 652 patients who underwent ROTA were included in the analysis, of whom 66 (10.1%) were classified as having CKD (eGFR < 60 mL/min/1.73 m^2^) and 586 (89.9%) as non-CKD. Diabetes mellitus (DM) was higher in the CKD group (78.8% vs. 54.2%, *p* < 0.001). Regarding cardiovascular history, prior peripheral vascular disease was more frequent in the CKD group (21.2% vs. 6.5%, *p* < 0.001), whereas prior myocardial infarction and prior coronary artery bypass grafting showed no statistically significant differences between groups. Clinical diagnosis of STEMI/NSTEMI was more included in the CKD group (39.4% vs. 26.0%, *p* = 0.021). Patients with CKD had a lower pre-procedural left ventricular ejection fraction compared with non-CKD patients (48.4 ± 14.0 vs. 54.0 ± 12.9, *p* = 0.001). Other vessel revascularization was performed more frequently in the CKD group (54.6% vs. 41.9, *p* = 0.049). Lastly, contrast volume was significantly less used in the CKD group (189.4 ± 95.6 vs. 214.1 ± 91.9, *p* = 0.044).

### 3.2. Primary Outcomes

Primary in-hospital outcomes are presented in Table 4. The composite outcome of CIN or in-hospital bleeding occurred more frequently in the CKD group compared with the non-CKD group (16.7% vs. 5.1%, *p* = 0.001). After adjustment for age, sex, smoking, hypertension, diabetes, clinical diagnosis of STEMI/NSTEMI, MVD, other vessel revascularization, procedural approach, and pre-procedural LV ejection fraction, CKD remained independently associated with the composite outcome (adjusted OR, 3.02; 95% CI, 1.36–6.69; *p* = 0.006). CIN occurred significantly more often in patients with CKD (15.2% vs. 1.7%, *p* < 0.001). In multivariable analysis using the same adjustment model, CKD was an independent predictor of CIN (adjusted OR, 7.18; 95% CI, 2.32–22.21, *p* < 0.001). In-hospital bleeding alone did not differ significantly between the non-CKD and CKD groups (3.0% vs. 3.6%, *p* > 0.999).

### 3.3. Secondary Outcomes

**Figure 2:**
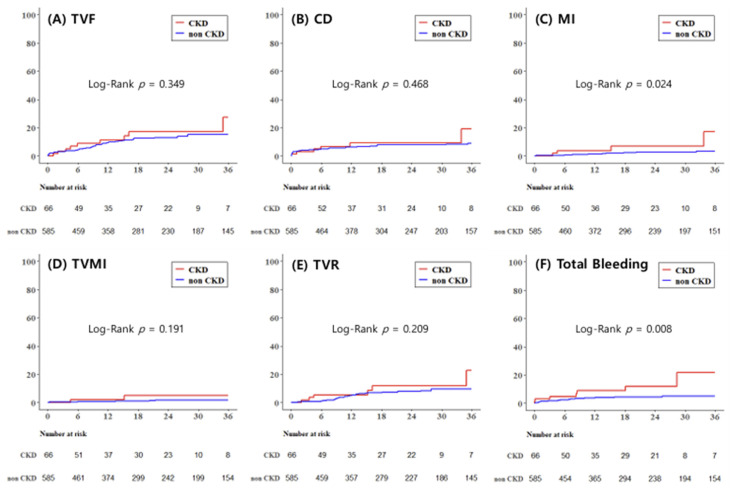
Kaplan–Meier curves for secondary outcomes during the follow-up period. (**A**) TVF, target vessel failure; (**B**) CD, cardiac death; (**C**) MI, myocardial infarction; (**D**) TVMI, target vessel myocardial infarction; (**E**) TVR, target vessel revascularization; (**F**) total bleeding.

3-year outcomes are summarized in Table 5. Median follow-up duration was 1.5 (IQR, 0.6–2.9) years. Myocardial infarction occurred more frequently in CKD patients (6.1% vs. 2.1%, log-rank *p* = 0.024), and CKD was independently associated with an increased risk of myocardial infarction in multivariable analysis (adjusted HR, 4.20; 95% CI, 1.23–14.32, log-rank *p* = 0.022). However, TVF, CD, TVMI, TVR, CVA, and ST were more common in the CKD group, though this did not reach statistical significance. Total bleeding events during follow-up occurred markedly more often in CKD patients (10.6% vs. 3.9%, log-rank *p* = 0.008), with CKD showing a strong independent association after multivariable adjustment (adjusted HR, 3.47; 95% CI, 1.41–8.56, log-rank *p* = 0.007). This association was primarily driven by minor bleeding events (adjusted HR, 7.65; 95% CI, 2.23–26.31, log-rank *p* = 0.001), whereas major bleeding did not differ significantly between two groups. Kaplan–Meier curves representing 3-year outcomes of TVF, CD, MI, TVMI, TVR, and total bleeding are shown in Figure 2.

### 3.4. Subgroup Analyses

**Figure 3:**
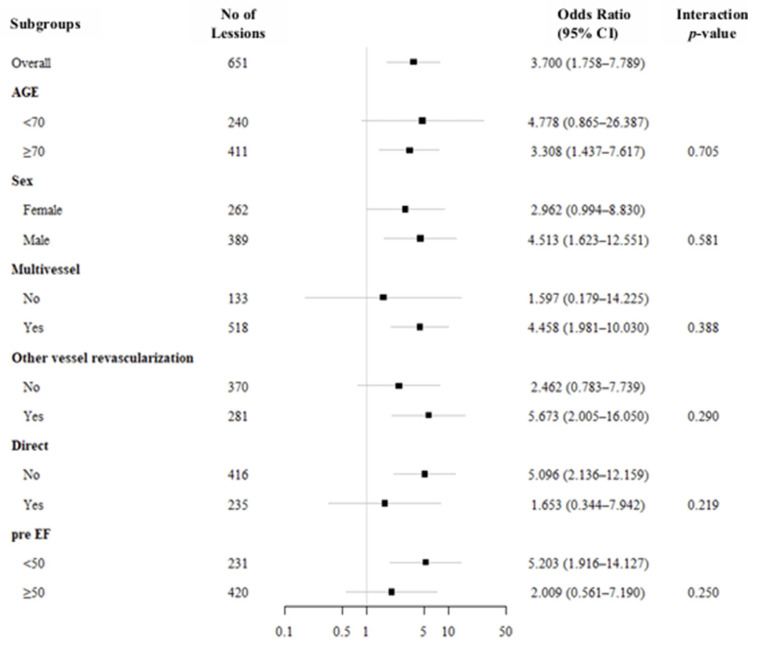
Forest plot for subgroup analysis.

Subgroup analyses showed that the association between CKD and the primary outcome (CIN or in-hospital bleeding) was generally consistent across all predefined subgroups (Figure 3). Although statistically significant associations were observed in certain subgroups, including patients aged ≥70 years, male patients, those with multivessel disease, those undergoing other vessel revascularization, those treated without a direct RA approach, and those with pre-procedural LVEF < 50%, none of the interaction tests reached statistical significance, indicating no evidence of effect modification. Given the wide confidence intervals in several subgroups, these findings should be interpreted with caution and considered exploratory.

## 4. Discussion

In this retrospective cohort study, CKD was independently associated with a significantly higher risk of CIN in patients undergoing ROTA. Subgroup analyses demonstrated that the association between CKD and the primary outcome was consistent across all subgroups. With respect to 3-year outcomes, myocardial infarction and total bleeding events occurred significantly more frequently in the CKD group, whereas major adverse cardiovascular events (MACEs), cerebrovascular accident (CVA), and stent thrombosis (ST) were numerically higher but did not reach statistical significance.

Coronary artery calcification in patients with CKD represents a critical determinant of both treatment strategy and long-term prognosis. Patients with CKD not only have a higher burden of coronary artery disease but also exhibit more advanced vascular calcification, often necessitating coronary intervention. Such interventions inherently require invasive procedures involving antithrombotic therapy with potential bleeding risk, as well as the unavoidable use of iodinated contrast agents, a well-established precipitant of acute kidney injury. In this context, CKD embodies a clinical phenotype intrinsically vulnerable to coronary procedures.

For patients with severe coronary calcification, preparation with RA is often required beyond conventional balloon angioplasty. Several studies have evaluated the clinical outcomes of RA-treated populations. Although not stratifying patients specifically by CKD status, prior investigations have identified low preoperative mean arterial pressure as an important predictor of CIN in RA-treated patients [24]. Moreover, studies examining plaque modification strategies have shown that patients requiring additional intervention for severe calcification experience higher rates of CIN compared with those undergoing less complex PCI [17]. Conversely, one study comparing RA and non-RA groups reported reduced MACE rates in the RA group during in-hospital and 2-year follow-up, suggesting potential procedural benefits in selected patients [25].

It is well established that CKD patients experience higher rates of CIN and bleeding complications following contrast-based invasive procedures compared with individuals with preserved renal function [26,27]. Several pathophysiological mechanisms may explain these observations. CKD patients are more susceptible to contrast-induced renal injury even at comparable contrast volumes due to impaired renal reserve and altered microvascular autoregulation. Although RA-treated patients are generally expected to require higher contrast volumes than non-RA PCI cases, the contrast volume used in our study was much lower than that reported in prior ROTA versus non-ROTA comparison study [28]. Importantly, in our study, the CKD group received significantly less contrast than the non-CKD group, yet experienced a markedly higher incidence of CIN. This discordance strongly supports that CKD itself plays a central role in the development of CIN, beyond the effect of contrast exposure. It suggests that procedural factors unique to RA play a critical role in renal injury. High-speed rotational ablation (above 150,000 rpm) can induce more pronounced microcirculatory dysfunction and microvascular stress compared to other plaque modification modalities like intravascular lithotripsy [29]. Moreover, the mechanical friction of the burr triggers platelet activation and endothelial injury markers [16]. The combined impact of RA-related mechanical stress, procedural complexity, and advanced coronary calcification may further deteriorate the severely limited microcirculatory reserve in patients with CKD [17,30]. Regarding bleeding risk, despite the interventional challenges associated with RA, in-hospital bleeding did not significantly differ between CKD and non-CKD groups. Although total bleeding events after 3-year follow-up were significantly higher in CKD patients, this difference was primarily driven by minor bleeding events. This is in line with prior large-scale data showing that radial access is associated with lower major bleeding compared with femoral access in RA procedures [31]; thus, the similar distribution of access sites between groups in our study may explain the comparable bleeding outcomes. Nevertheless, given the significantly increased long-term bleeding risk observed in CKD patients, individualized therapeutic planning that incorporates bleeding propensity remains important. The biological basis for increased bleeding in CKD is well recognized. CKD is associated with platelet dysfunction, endothelial abnormalities, and uremia-related coagulopathy, all contributing to an elevated bleeding tendency [32]. Current guidelines also recognize CKD as a major determinant of bleeding risk and recommend a consideration of early de-escalation or shortening of dual antiplatelet therapy when clinically appropriate [33,34,35]. Furthermore, the preferential use of polymer-free or bioabsorbable polymer stents may facilitate shorter durations of dual antiplatelet therapy without compromising ischemic protection in this high-risk population [36].

Our study has several distinctive features. Unlike previous studies, we specifically assessed the impact of CKD within a homogeneous population undergoing RA, a procedural population for which data remain limited in the literature. In this setting, we demonstrate that CKD remains a strong independent predictor of contrast-induced nephropathy despite lower contrast exposure, suggesting that factors beyond contrast alone contribute to renal injury. RA-related mechanical stress may further aggravate the already compromised microvascular reserve in CKD patients. Moreover, clinical outcomes of our study show a divergent pattern with preserved revascularization-related endpoints but increased susceptibility to myocardial infarction and bleeding. These findings therefore extend the current knowledge of CKD-related risk to this complex interventional setting and may support more individualized procedural and post-procedural strategies.

Consistent with our results, there are several previous studies supporting RA for the CKD population with acceptable procedural success and safety outcomes. One supported the use of RA with contemporary DES as a lesion preparation option when meticulous procedural planning is applied in CKD [37]; another study of patients with advanced CKD, including those with end-stage renal disease on dialysis, reported no significant difference in acute procedural complications according to renal function and emphasized the feasibility of RA in advanced CKD [38].

Moreover, our findings support a more individualized interventional strategy in CKD patients undergoing RA. In those with multivessel disease, an early consideration of staged PCI may help reduce contrast burden. In elderly CKD patients, thorough pre-procedural imaging assessment and strategic planning—including contrast minimization and the use of a direct ROTA approach to reduce procedural time—may prevent renal injury. In recent data regarding the IVUS-guided minimal-contrast RA approach, CIN occurred infrequently and procedural safety was preserved, supporting the concept that imaging-guided RA can be leveraged to reduce renal risk in vulnerable patients [39]. Although acute complications may not be entirely avoidable, appropriate early management may allow CKD patients to achieve long-term outcomes comparable to those without CKD, supporting the feasibility of RA in this high-risk population.

This study has several limitations. First, the retrospective, observational study design precludes causal inference and may allow residual confounding. Second, propensity score matching was not performed due to the limited number of CKD patients, which could have resulted in the substantial loss of cases and events. Instead, we applied multivariable adjustment and sensitivity analyses to mitigate potential bias. Third, though the sample size was not large, the cohort consisted exclusively of patients undergoing RA, making the study adequately powered within this high-risk population. Fourth, although time-to-event analyses accounted for variable follow-up durations, the relatively limited proportion of patients with complete 3-year follow-up may reduce the precision of long-term outcome estimates. Fifth, patients with end-stage renal disease on dialysis were excluded because CIN was a primary endpoint, which limits generalizability but was necessary to accurately evaluate contrast-related renal injury. Sixth, procedural strategies and contrast use were operator-dependent, and minor bleeding events may have been underreported; however, key clinical variables were incorporated into adjusted analyses. Seventh, we did not account for the potential impact of contemporary pharmacologic therapies, such as sodium–glucose cotransporter-2 inhibitors and mineralocorticoid receptor antagonists, which have recently gained prominence in both cardiology and nephrology. Given the established interaction between chronic kidney disease, heart failure, and CIN [40], future studies incorporating these therapeutic factors and focusing on patients with concomitant CKD and heart failure may provide additional insight into risk stratification in this high-risk population. Eighth, although severe coronary artery calcification (CAC) is a recognized independent predictor of CIN risk in CKD patients [30], we were unable to quantitatively assess CAC scores or utilize coronary CT to characterize the extent of calcification in this retrospective analysis. Future studies incorporating both coronary and renal artery calcification assessments may provide a more comprehensive understanding of the systemic vascular vulnerability and renal risk profiles in this high-risk population.

In patients undergoing RA for severely calcified coronary lesions, CKD independently increases the risk of CIN and long-term bleeding, as well as ischemic events such as MI. However, considering comparable revascularization outcomes but not fully satisfying ischemic outcomes, RA could still be one of the treatment strategies in CKD patients when early complications are carefully managed. These findings emphasize the need for contrast-minimizing procedural protocols, thoughtful lesion preparation planning, and tailored antiplatelet regimens in CKD patients undergoing complex coronary intervention.

## 5. Conclusions

In patients undergoing RA for severely calcified coronary lesions, CKD is a robust independent risk factor for CIN and long-term bleeding complications, particularly minor bleeding. However, except for MI, the revascularization and ischemic outcomes showed no statistically different results between CKD and non-CKD groups. These findings suggest that, with optimized peri-procedural strategies to minimize contrast burden and bleeding complications, RA could be considered as a viable treatment option in CKD patients.

## Figures and Tables

**Table 1:** Baseline characteristics.

	Non CKD	CKD	*p*-Value
	*n* = 586	*n* = 66	
Age	72.0 ± 9.6	74.3 ± 8.3	0.069
Sex	351 (60.0)	38 (57.6)	0.703
BMI	24.2 ± 3.9	24.1 ± 3.6	0.874
Systolic BP	131.9 ± 24.1	133.5 ± 18.2	0.581
Diastolic BP	74.8 ± 12.5	74.3 ± 10.9	0.737
Smoking	111 (19.0)	7 (10.6)	0.094
HTN	446 (76.2)	57 (86.4)	0.063
DM	317 (54.2)	52 (78.8)	<0.001
Dyslipidemia	301 (51.5)	33 (50.0)	0.823
CKD (no dialysis)	0 (0.0)	66 (100.0)	<0.001
LV Ejection Fraction	54.0 ± 12.9	48.4 ± 14.0	0.001
Medical History			
PCI	136 (23.3)	19 (28.8)	0.317
CABG	24 (4.1)	4 (6.1)	0.515
MI	64 (10.9)	9 (13.6)	0.511
CVA	93 (15.9)	10 (15.2)	0.875
PVD	38 (6.5)	14 (21.2)	<0.001
Chronic lung disease	34 (5.8)	8 (12.1)	0.061
Heart failure	73 (12.5)	12 (18.2)	0.192
Atrial fibrillation	42 (7.2)	10 (15.2)	0.024
Clinical_diagnosis (STEMI/NSTEMI)	152 (26.0)	26 (39.4)	0.021

CKD, chronic kidney disease; BMI, body mass index; BP, blood pressure; HTN, hypertension; DM, diabetes mellitus; LV, left ventricle; PCI, percutaneous coronary intervention; CABG, coronary artery bypass graft; MI, myocardial infarction; CVA, cerebrovascular accident; PVD, peripheral vascular disease; NSTEMI, non-ST segmental elevation myocardial infarction; STEMI, ST segmental elevation myocardial infarction.

**Table 2:** Laboratory analyses and medications.

	Non CKD	CKD	*p*-Value
	*n* = 586	*n* = 66	
Laboratory analyses			
Hb	12.6 ± 2.3	11.1 ± 1.8	<0.001
Platelet	220.8 ± 65.5	219.8 ± 93.0	0.910
Triglyceride	119.0 ± 74.2	123.1 ± 66.0	0.686
Total cholesterol	146.5 ± 40.1	134.6 ± 35.3	0.030
LDL cholesterol	85.2 ± 39.3	75.6 ± 31.9	0.079
HDL cholesterol	47.1 ± 14.7	40.2 ± 14.3	<0.001
Hs-CRP	2.6 ± 10.6	2.5 ± 6.8	0.971
HbA1c	6.7 ± 1.6	7.4 ± 1.5	0.002
Medication			
NOAC	21 (3.6)	5 (7.6)	0.171
DAPT	559 (95.6)	61 (92.4)	0.230
Aspirin	571 (97.6)	62 (93.9)	0.099
P2Y12_inhibitor	574 (98.1)	66 (100.0)	0.614
Cilostazol	73 (12.5)	6 (9.1)	0.424
Beta-blocker	392 (67.0)	45 (68.2)	0.848
ACEI or ARB	377 (64.4)	39 (59.1)	0.391
Statin	552 (94.4)	62 (93.9)	0.782

Hb, hemoglobin; LDL, low-density lipoprotein; HDL, high-density lipoprotein; Hs-CRP, high-sensitivity C-reactive protein; HbA1c, hemoglobin A1c; NOAC, new oral anticoagulant; DAPT, dual antiplatelet therapy; P2Y12_inhibitor, antiplatelet agents targeting the P2Y12 receptor (e.g., clopidogrel, prasugrel, ticagrelor); ACEI/ARB, angiotensin converting enzyme inhibitor/angiotensin II receptor blocker.

**Table 3:** Lesion and procedural characteristics.

	Non-CKD	CKD	*p*-Value
	*n* = 586	*n* = 66	
Lesion Classification (B2/C)	549 (93.9)	62 (93.9)	>0.999
Vessel disease			
1VD	124 (21.2)	9 (13.6)	0.312
2VD	187 (32.0)	25 (37.9)	
3VD	274 (46.8)	32 (48.5)	
Multivessel disease (MVD)	461 (78.8)	57 (86.4)	0.149
LM disease	86 (14.7)	11 (16.7)	0.671
IVUS	303 (51.8)	37 (56.1)	0.511
Direct	211 (36.1)	24 (36.4)	0.962
Other vessel revascularization	245 (41.9)	36 (54.6)	0.049
Procedural approach			
Radial	291 (49.7)	31 (47.0)	0.669
Femoral	294 (50.3)	35 (53.0)	
Procedure success	555 (94.9)	64 (97.0)	0.762
Technical success	562 (96.1)	66 (100.0)	0.155
Contrast (mL)	214.1 ± 91.9	189.4 ± 95.6	0.044
Procedural time	78.0 ± 51.4	86.5 ± 56.4	0.211
Radiation dose	3811.6 ± 3643.3	4354.1 ± 2684.3	0.574
Rotational atherectomy			
Size of burr (start)	1.5 ± 0.2	1.5 ± 0.2	0.951
Size of burr (max)	1.5 ± 0.6	1.5 ± 0.2	0.795
Number of burrs	1.2 ± 0.4	1.2 ± 0.5	0.991
Stent			
Stent diameter	3.0 ± 0.4	3.0 ± 0.3	0.384
Total number of stents	2.5 ± 1.3	2.6 ± 1.3	0.377
Total length of stent	69.6 ± 37.7	74.3 ± 38.4	0.343
Peri-procedural complication			
Coronary dissection	192 (32.8)	19 (28.8)	0.507
Coronary perforation	21 (3.6)	2 (3.0)	>0.999
Urgent intervention for tamponade	3 (0.5)	0 (0.0)	>0.999
Temporary pacemaker	39 (6.7)	6 (9.1)	0.442
Periprocedural MI	54 (9.2)	10 (15.2)	0.126
In-hospital outcome			
In-hospital death	18 (3.1)	2 (3.0)	>0.999
In-hospital CVA	2 (0.3)	0 (0.0)	>0.999
Urgent CABG	2 (0.3)	0 (0.0)	>0.999
Urgent PCI	5 (0.9)	1 (1.5)	0.475

LM, left main; IVUS, intravascular ultrasound sonography; MI, myocardial infarction; CVA, cerebrovascular accident; CABG, coronary artery bypass graft; PCI, percutaneous coronary intervention; Lesion Classification (B2/C), according to the ACC/AHA lesion classification; B2/C lesions indicate complex coronary anatomy, including features such as long lesion length, severe calcification, tortuosity, or chronic total occlusion.

**Table 4:** Primary outcomes.

Primary Outcome	Non CKD	CKD	*p*-Value	Univariate				Multivariate *			
	*n* = 586	*n* = 66		OR	95%CI (Lower–Upper)		*p*-Value	OR	95%CI (Lower–Upper)		*p*-Value
Composite ^a^	30 (5.1)	11 (16.7)	0.001	3.700	1.758	7.789	<0.001	3.019	1.363	6.685	0.006
CIN	10 (1.7)	10 (15.2)	<0.001	10.269	4.099	25.727	<0.001	7.184	2.324	22.210	<0.001
In-hospital bleeding	21 (3.6)	2 (3.0)	>0.999	0.839	0.192	3.662	0.816	0.779	0.170	3.565	0.747

* Adjusted for age, sex, smoking, comorbidities of hypertension and diabetes mellitus, clinical diagnosis of STEMI/NSTEMI, multivessel disease, other vessel revascularization, procedural approach, pre-procedural left ventricular ejection fraction, and contrast volume. ^a^ Defined as the composite of CIN and in-hospital bleeding. Abbreviations: CIN, contrast induced nephropathy; OR, odds ratio; CI, confidence interval.

**Table 5:** Secondary outcomes.

Secondary Outcome (3-Year Outcome)	Non-CKD	CKD	*p*-Value	Log-Rank *p*-Value	Univariate				Multivariate (Firth’s Penalized Likelihood) *			
	*n* = 586	*n* = 66			HR	95%CI (Lower–Upper)		*p*-Value	HR	95%CI (Lower–Upper)		*p*-Value
TVF	63 (10.8)	9 (13.6)	0.481	0.349	1.395	0.693	2.807	0.351	1.372	0.671	2.806	0.386
AD	55 (9.4)	9 (13.6)	0.273	0.210	1.565	0.772	3.171	0.214	1.381	0.673	2.833	0.379
CD	41 (7.0)	6 (9.1)	0.461	0.468	1.373	0.582	3.236	0.469	1.345	0.566	3.193	0.502
MI	12 (2.1)	4 (6.1)	0.069	0.024	3.417	1.097	10.645	0.034	4.201	1.232	14.324	0.022
TVMI	7 (1.2)	2 (3.0)	0.229	0.191	2.735	0.568	13.175	0.210	4.304	0.718	25.806	0.110
RR	50 (8.6)	6 (9.1)	0.881	0.723	1.165	0.499	2.719	0.724	0.994	0.422	2.339	0.988
TVR	35 (6.0)	6 (9.1)	0.291	0.209	1.733	0.727	4.128	0.215	1.540	0.629	3.768	0.345
TLR	31 (5.3)	4 (6.1)	0.772	0.632	1.289	0.454	3.660	0.633	1.192	0.415	3.420	0.744
NLR	25 (4.3)	3 (4.6)	0.756	0.756	1.209	0.365	4.010	0.756	1.299	0.394	4.282	0.667
CVA	8 (1.4)	3 (4.6)	0.091	0.054	3.419	0.906	12.913	0.070	3.542	0.879	14.275	0.075
ST	5 (0.9)	1 (1.5)	0.475	0.603	1.756	0.205	15.028	0.607	2.270	0.259	19.898	0.459
Total bleeding	23 (3.9)	7 (10.6)	0.025	0.008	2.983	1.275	6.978	0.012	3.474	1.410	8.561	0.007
Minor bleeding	9 (1.5)	5 (7.6)	0.009	<0.001	5.776	1.918	17.396	0.002	7.651	2.225	26.310	0.001
Major bleeding	9 (1.5)	1 (1.5)	>0.999	0.940	1.082	0.137	8.577	0.940	1.797	0.253	12.775	0.558

* Adjusted for age, sex, smoking, comorbidities of hypertension and diabetes mellitus, clinical diagnosis of STEMI/NSTEMI, multivessel disease, other vessel revascularization, procedural approach, pre-procedural left ventricular ejection fraction, and contrast volume; using Firth’s penalized likelihood Cox regression to account for small event counts. Abbreviations: TVF, target vessel failure; AD, all-cause death; CD, cardiac death; MI, myocardial infarction; TVMI, target vessel spontaneous MI; RR, repeat revascularization; TVR, target vessel revascularization; TLR, target lesion revascularization; NLR, neutrophil-to-lymphocyte ratio; CVA, cerebrovascular accident; ST, stent thrombosis; HR, hazard ratio; CI, confidence interval; TVF is defined as composite of CD, TVMI, and TVR.

## Data Availability

The data included in this manuscript cannot be shared publicly, due to the need to protect the privacy of the included subjects. Data may be shared upon reasonable request to the corresponding authors.

## Abbreviations

The following abbreviations are used in this manuscript:

CABG	Coronary artery bypass graft
CD	Cardiac death
CIN	Contrast-induced nephropathy
CKD	Chronic kidney disease
CVA	Cerebrovascular accident
LVEF	Left ventricle ejection fraction
MVD	Multivessel disease
PCI	Percutaneous coronary intervention
RA	Rotational atherectomy
ST	Stent thrombosis
RR	Repeat revascularization
TVF	Target vessel failure
TVMI	Target vessel spontaneous myocardial infarction
TVR	Target vessel revascularization
